# Impact of Conformational Substates and Energy Landscapes on Understanding Hemoglobin Kinetics and Function

**DOI:** 10.1007/s10867-021-09588-3

**Published:** 2021-11-11

**Authors:** William A. Eaton

**Affiliations:** Laboratory of Chemical Physics, National Institute of Diabetes and Digestive and Kidney Diseases, National Institutes of Health, 5/104, Bethesda, MD 20892-0520 United States

**Keywords:** Myoglobin, Hemoglobin, Protein kinetics, Allostery, Cooperativity, Kramers theory, Internal friction

## Abstract

Hans Frauenfelder’s discovery of conformational substates in studies of myoglobin carbon monoxide geminate rebinding kinetics at cryogenic temperatures (Austin RH, Beeson KW, Eisenstein L, Frauenfelder H, & Gunsalus IC (1975) Dynamics of Ligand Binding to Myoglobin. *Biochemistry* 14(24):5355–5373) followed by his introduction of energy landscape theory with Peter Wolynes (Frauenfelder H, Sligar SG, & Wolynes PG (1991) The Energy Landscapes and Motions of Proteins. *Science* 254(5038):1598–1603) marked the beginning of a new era in the physics and physical chemistry of proteins. Their work played a major role in demonstrating the power and importance of dynamics and of Kramers reaction rate theory for understanding protein function. The biggest impact of energy landscape theory has been in the protein folding field, which is well-known and has been documented in numerous articles and reviews, including a recent one of my own (Eaton WA (2021) Modern Kinetics and Mechanism of Protein Folding: a Retrospective. *J. Phys. Chem. B.* 125(14):3452–3467). Here I will describe the much less well-known impact of their modern view of proteins on both experimental and theoretical studies of hemoglobin kinetics and function. I will first describe how Frauenfelder’s experiments motivated and influenced my own research on myoglobin, which were key ingredients to my work on understanding hemoglobin.

## Introduction

**Fig. 1:**
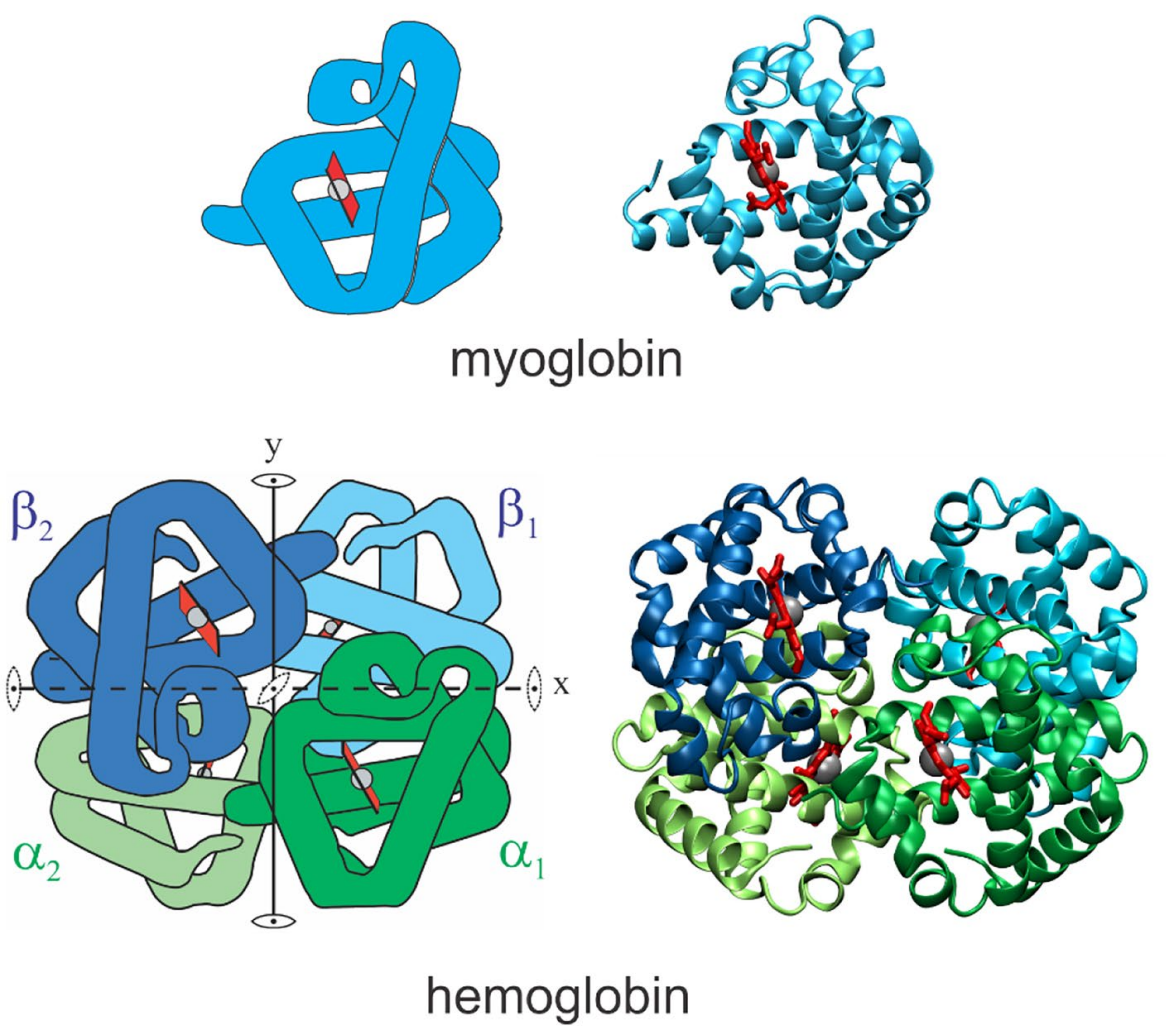
Schematic structures of myoglobin and hemoglobin. Images on right show polypeptide backbone and hemes. Images on left are adapted from Dickerson and Geis [3]. The straight and almost straight segments of the Dickerson and Geis images consist of α-helices. The heme groups are red and the iron atom to which oxygen binds is the grey sphere. In both molecules, the iron atom is covalently bonded to 4 nitrogens of the planar porphyrin ring and the imidazole nitrogen of a histidine side chain of the globin. Hemoglobin has one exact two-fold symmetry axis (y) that interchanges α_1_β_1_ and α_2_β_2_ and, because of the similarity in structure of the α and β subunits, has 2 pseudo-two-fold symmetry axes (x and z)

Understanding the physics of proteins for the past 50 years have been strongly influenced by studies of myoglobin and hemoglobin, in large part because these were the first proteins for which three-dimensional structures at atomic resolution were determined. The physiological function of myoglobin is to store oxygen in muscle, while hemoglobin in the red cells of blood transports oxygen from the lungs to the tissues. Oxygen binds to the iron atom of the single heme of myoglobin and to the irons of the 4 hemes of hemoglobin. The X-ray crystallographic structure determination of these molecules earned John Kendrew and Max Perutz the 1962 Nobel Prizes in Chemistry. As shown in Fig. 1, the 4 subunits of hemoglobin are primarily α-helical with individual subunits that are similar in structure to myoglobin. The famous biological physicist, John J. Hopfield, called myoglobin the “hydrogen atom of biology”, which makes hemoglobin the hydrogen molecule of biology. As the protein of blood, hemoglobin is the most readily available of all proteins and, consequently, has been the subject of chemical and physical studies since the nineteenth century. Another reason that myoglobin and hemoglobin have been so extensively investigated is that they can be studied by almost any physical or spectroscopic method—a major attractor to physical scientists to study these molecules. To paraphrase one of President Harry S. Truman’s quotes: “The only thing new in biophysical science is the history of myoglobin and hemoglobin you do not know,” which is only a slight exaggeration. Modern studies of myoglobin began with the experiments of Hans Frauenfelder [1], while modern studies of hemoglobin began with the theoretical model of Jacque Monod, Jeffries Wyman, and Jean-Pierre Changeux [2].

**Fig. 2:**
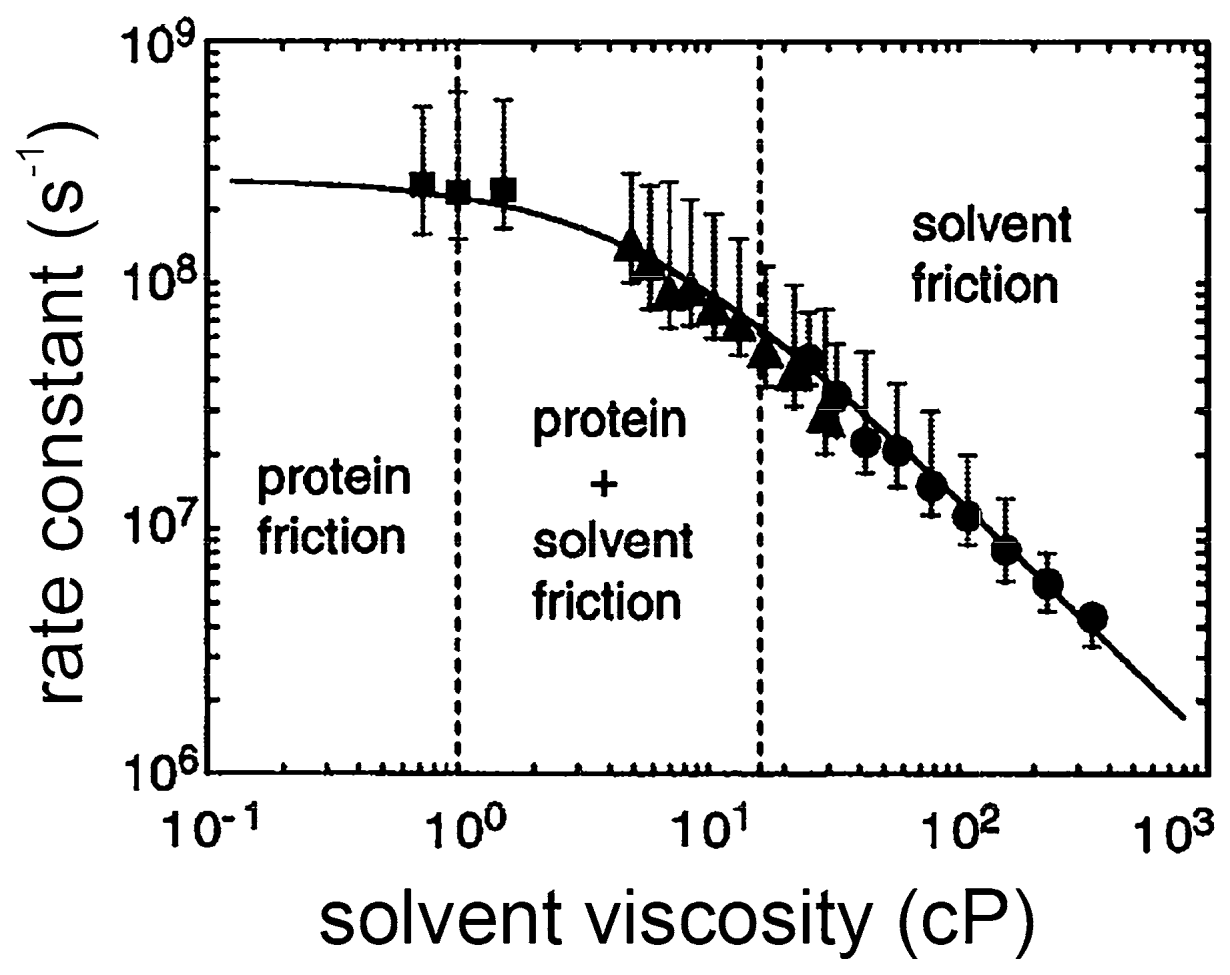
Rate constant for conformational change of myoglobin following photodissociation of CO as a function of solvent viscosity [18]. The smooth curve is a fit to the data using the equation: *k* = C/(σ+η)exp(-*E*_0_/*kT*), where the term with units of viscosity, σ (~ 4 cP), is the contribution to the total friction from the protein friction

**Fig. 3:**
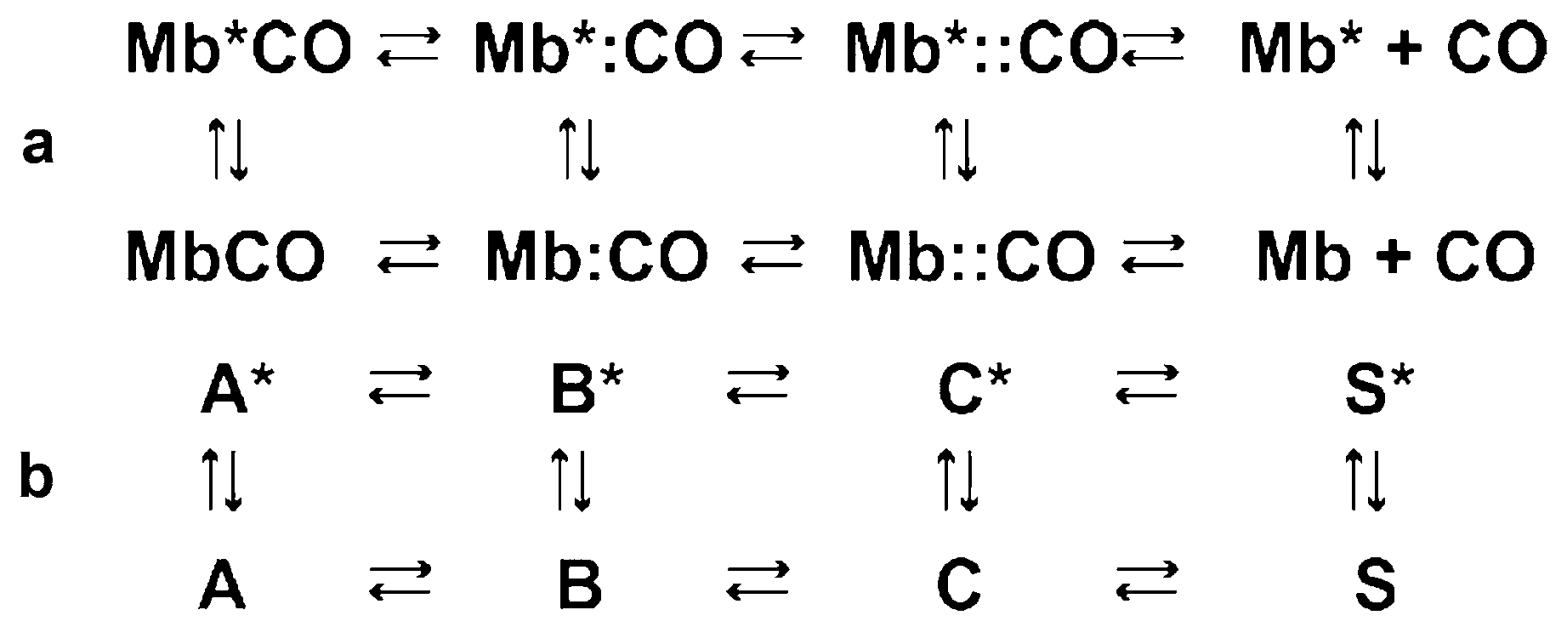
Kinetic model for CO rebinding to myoglobin at room temperature. (**a**) The model contains 2 geminate states and 2 distributions of conformational substates indicated by Mb* and Mb. (**b**) The same mechanism using Frauenfelder notation

## Discovery of geminate rebinding in myoglobin and hemoglobin at room temperature

In his seminal article in 1975, Hans Frauenfelder reported results of his student Robert Austin on measurements in glycerol-water glasses of carbon monoxide (CO) rebinding kinetics to myoglobin from 2 μs to 1000 s following CO photodissociation in the temperature range 40 K to 160 K [1]. At the lowest temperatures they observed kinetics corresponding to a very broad distribution of CO rebinding rates, which they interpreted as arising from geminate rebinding to different protein conformations that do not interconvert because of high energy barriers separating them (geminate rebinding is binding of the CO to the same heme from which it was photodissociated). Austin et al. called these non-interconverting conformations “conformational substates”. The discoveries in this paper marked the beginning of a new era in the study of the physics and physical chemistry of proteins.

The experiments of Austin et al*.* motivated attempts by many research groups to measure geminate rebinding at room temperature, which was expected to require much better time resolution than microseconds in Austin et al. While the low temperature measurements of Austin et al*.* exposed the underlying physics of geminate ligand rebinding, measurements at ambient temperature were necessary for making direct connection to the physiological function of heme-containing proteins. The first attempt was made by Charles Shank, the inventor of femtosecond laser pulses. He used sub-picosecond laser pulses to photodissociate both CO and oxygen (O_2_) from hemoglobin, myoglobin’s big brother (Fig. 1), but only monitored absorption at a single wavelength (615 nm) [4]. Shank observed a rapid (0.5 ps) increase in absorption that he assumed to result from photodissociation of the CO to form deoxyHb. He also observed an intensity change with a 2.5 ps lifetime after exciting deoxyHb and oxyHb, which he attributed to the decay of excited electronic states. An alternative interpretation was suggested by molecular dynamics simulations and the experiments of Philip Anfinrud and coworkers [5, 6]. Anfinrud observed cooling of the heme with a time constant of 6 ps in deoxyMb following heating by the energy in a femtosecond laser pulse. The second attempt was made by Robin Hochstrasser and coworkers using 10 picosecond pulses to photodissociate both CO and O_2._ They measured absorption at multiple wavelengths to obtain spectra that could more readily identify photoproducts [7]. Since the time between the excitation and probe pulses was determined by the velocity of light and the distance traveled by the probe pulse before entering the sample, the delay line employed in the Hochstrasser experiments only permitted the kinetics to be studied up to 680 psec. Photodissociation of CO resulted in the immediate appearance of the deoxyHb spectrum, as expected from Shank’s experiment. The spectral changes following excitation of oxyhemoglobin were less definitive and, like Shank, were interpreted in terms of transitions between electronically excited states. Using nanosecond laser pulses, where there was no limitation on the time between excitation and probe pulses, Duddell et al*.* were the first to observe CO geminate rebinding at ambient temperature [8]. Their experiments at 277 K on hemoglobin showed a geminate yield of ~ 50% and a relaxation time of ~150 ns. Duddell et al. also observed geminate rebinding of oxygen to myoglobin with a geminate yield of 27% at 298 K and 55 ns relaxation time [9]. Geminate rebinding of O_2_ to hemoglobin was first observed by Hochstrasser and coworkers at 278 K, which showed ~ 40% geminate yield and relaxation time of ~ 200 ps [10].

Assuming a temperature independent activation energy, extrapolation of the geminate kinetics at low temperature to room temperature predicted a CO geminate yield of less than 1.3% for myoglobin [11]. So, measurement of geminate rebinding of CO in myoglobin at room temperature was expected to be quite challenging. To do so required a high precision nanosecond absorption spectrometer, as developed by James Hofrichter and Eric Henry [12]. They found that the geminate yield at room temperature is only 4% with a relaxation time of ~ 300 ns [13]. A comparison of rate coefficients for CO and O_2_ suggested that the higher geminate yield for O_2_ arises from the much faster reaction of O_2_ with the heme iron. This difference in rates has been explained by differences in steric effects [14, 15]. Differences in spin state of CO (S = 1) and O_2_ (S = 3) reacting with the heme iron (S = 5) may also play a role [7].

## Discovery of internal friction from solvent viscosity studies and of distributed geminate rebinding rates in a room temperature glass

One of the hallmarks of energy landscape theory is the description of kinetics as diffusion on a low-dimensional free energy surface [16]. As in Arrhenius theory, the reaction rate is determined primarily by the exponential factor. However, the pre-exponential factor is simply not a constant or the *k*_*B*_*T*/*h* factor as in Eyring’s gas phase transition state theory but depends on the friction opposing atomic motions, as described by Kramers. Frauenfelder and coworkers were the first to apply Kramers theory to protein reactions [11]. They showed that the unimolecular time constants for CO transition between geminate states or escaping from the protein into the liquid solvent exhibits a fractional viscosity dependence. Beece et al. proposed that each substate has an open and closed conformation, with the transition between them that is affected by (“slaved” to) the solvent viscosity. My Laboratory of Chemical Physics colleague, Robert Zwanzig, showed that a fractional viscosity dependence can arise from a fluctuating barrier height [17].

Frauenfelder’s viscosity studies motivated his former student, Anjum Ansari, to look at conformational relaxation directly by nanosecond resolved spectroscopy following photodissociation of CO, where photoselection effects from rotational diffusion of myoglobin were eliminated by using the isotropically averaged spectra obtained from measurements in orthogonally polarized light [18–21]. By applying singular value decomposition to analyze the transient spectra, Ansari and coworkers discovered spectral changes of the unliganded heme in the time window from ~ 10 ns to ~ 10, μs, which they interpreted as resulting from a non-exponential conformational relaxation of the protein because of the close similarity to the unliganded spectral changes observed for the well-known conformational change in hemoglobin discussed below [18, 21]. They discovered that the conformational relaxation rate depends inversely on the first power of the solvent viscosity at high viscosities, as expected from Kramers theory in the high friction limit. At low viscosities, the relaxation rate becomes independent of solvent viscosity, which they attributed to dominance of internal friction from protein atom–atom collisions (Fig. 2) [18, 21]. The data in (Fig. 2) were well fit with a simple equation that assumes the protein and solvent friction can be summed, so that the total viscosity is the sum of the solvent viscosity (η) and a viscosity (σ) corresponding to internal protein friction [22].

Ansari et al*.* built a kinetic model with two geminate states (Fig. 3), instead of the 3 geminate states of Frauenfelder’s model [21]. Each geminate state corresponds to a different location for the CO inside the protein, from which it can rebind to the heme before escaping into the solvent. Ansari’s measured non-exponential conformational relaxation from Mb* to Mb that slows geminate rebinding was incorporated into the model (the significance of the asterisk * is discussed later). Conformational relaxation that slows geminate rebinding was previously proposed by Noam Agmon and John Hopfield. In their model, which considered only a single geminate state, the protein diffuses along a protein reaction coordinate with the barrier to ligand rebinding dependent on the protein coordinate position [23]. It was also proposed, but not measured, in Steinbach et al., which contained Frauenfelder’s most comprehensive modeling of CO rebinding kinetics [24]. The location of the CO in geminate states was subsequently elucidated by picosecond resolved X-ray crystallography by Anfinrud, Brunori and coworkers [25, 26].

**Fig. 4:**
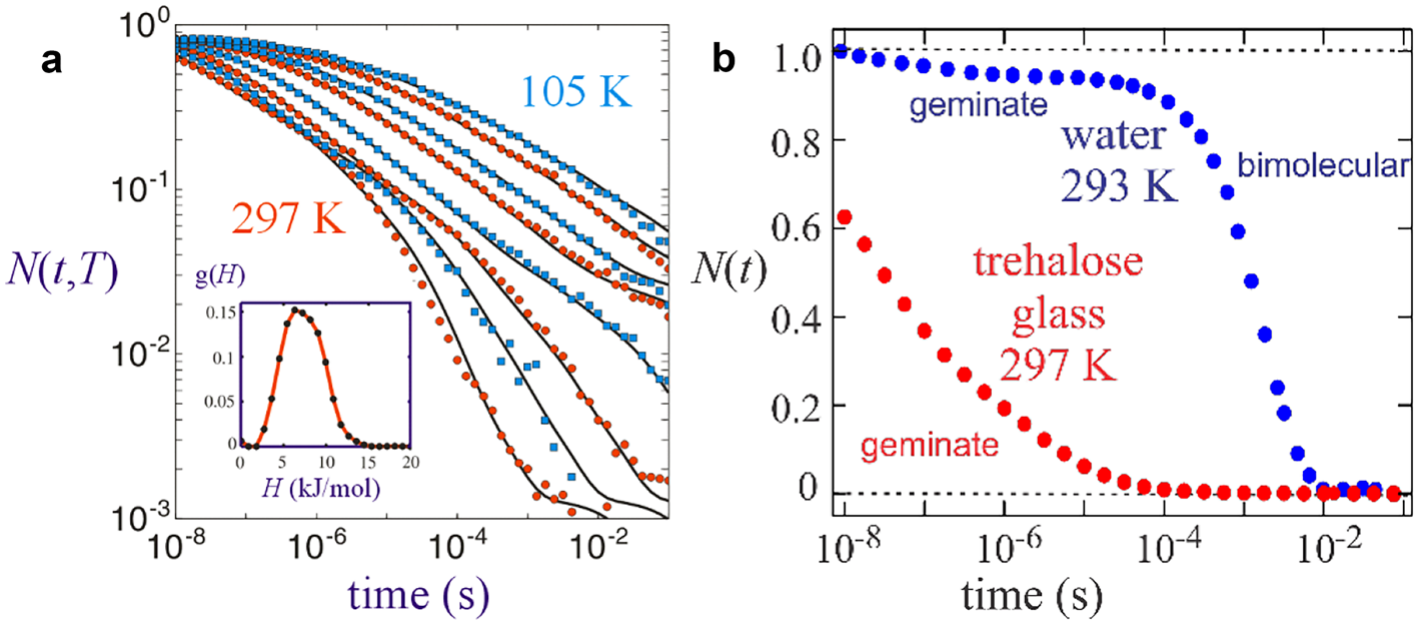
The fraction of myoglobin molecules *N*(*t*) remaining unliganded at time *t* after CO photolysis. (**a**) rebinding of CO in a trehalose glass between 105 and 297 K [30]. The inset shows the activation enthalpy distribution that best fits the rate distribution (continuous lines) assuming 32 conformational substates. (**b**) Comparison of CO rebinding in trehalose glass and water [21]. The geminate yield is only about 4% because the escape of the CO from the protein is about 20-times faster than the geminate rebinding rate, so 96% of the photdissociated CO molecules rebind from the solvent in a bimolecular process, while in the trehalose glass all CO molecules remain in the protein to geminately rebind

An important result of Ansari’s analysis was the proposal that the geminate rate distribution does not result from insufficient kinetic energy at cryogenic temperature to cross high energy barriers. Instead, as also suggested in Frauenfelder’s earlier viscosity paper, it is due to the lack of interconversion of conformational substates from the high viscosity of the glycerol-water glass that determines the pre-exponential factor in Kramers theory [18, 21]. Ansari’s proposal received strong support from the work of Stephen Hagen who studied CO rebinding from 105 K to 297 K in a glass of trehalose that has a viscosity of ~ 10^8^ centipoise at 297 K (Fig. 4) [27]. It was also supported by molecular dynamics calculations by Karplus using a Nose–Hoover thermostat, in which the temperature of the protein and solvent can be different [28]. Hagen et al. found distributed rebinding kinetics at 297 K (Fig. 4a). Hagen’s data were well fit at every temperature with a distribution of activation enthalpies g(*H*) shown in the insert to Fig. 4a using

$$N(t) = \sum\limits_i {g({H_i})\exp \left( { - k({H_i})t} \right)} = \sum\limits_i {g({H_i})\exp \left( { - \left( {A\exp \left( { - {H_i}/{k_B}T} \right)} \right)t} \right)}$$

where *N*(*t*) is the time (*t*)-dependent survival probability, *g*(*H*_*i*_) is the enthalpy (*H*) distribution of substates *i*, *k*(*H*_*i*_) is the rate coefficient distribution that results from the enthalpy distribution, *A* is the Arrhenius prefactor, *k*_*B*_ is Boltzmann’s constant and *T* is the absolute temperature. Hagen’s experiments showed that high solvent viscosity prevents interconversion of conformational substates and in doing so prevents the conformational relaxation of the protein that slows geminate rebinding. Frauenfelder subsequently proposed that, in addition to bulk solvent viscosity (called α fluctuations), there is an additional effect on conformational substate dynamics from fluctuations of the solvent molecules that hydrate the protein surface (called β fluctuations) [29].

**Fig. 5:**
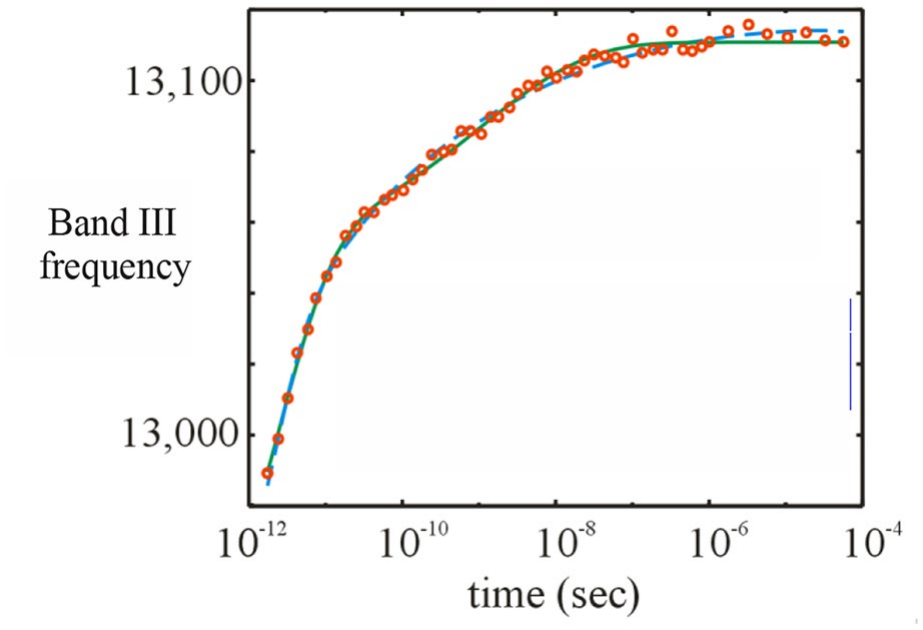
Frequency shift of the conformationally-sensitive 760 nm porphyrin π to iron d charge transfer band [34] following photodissociation of CO from myoglobin measured by Anfinrud and coworkers (points) [33]. The curves through the data are the fits from the model of Hagen et al. in two cases for the kinetic connectivity of conformational substates between Mb* and Mb [35]

The initial rate at 297 K for CO geminate rebinding to Mb* in the glass is given by

$$\frac{{dN{{(t)}_{t \to 0}}}}{dt} = - \sum\limits_i {g({H_i})} k({H_i})$$

and is 5 × 10^7^ s^−1^, 2500-fold faster than the measured geminate rebinding rate in aqueous solution of 2 × 10^4^ s^−1^ [21]. The much faster rate in the glass is due to the high viscosity preventing the conformation from relaxing to the much more slowly reacting conformation that is almost complete (Fig. 5) in liquid aqueous solution prior to the onset of geminate rebinding (Fig. 4b). (In an elegant analysis Hagen showed that the spectral changes in the glass are not due to conformational relaxation but due to kinetic hole burning in which conformational substates that bind with different rates also have different spectra). The difference in CO geminate rebinding rates to the high and low oxygen affinity conformations of hemoglobin discussed below is only ~ 30-fold [31, 32], where there is a much larger difference in protein structure. Given the barely observable differences in structure between MbCO and Mb (~ 0.2 Å rms, see Fig. 1 in ref. [21]), the structural origin for the wide distribution of geminate rebinding rates for conformational substates at cryogenic temperatures and myoglobin in glass at room temperature is yet to be explained.

## Theoretical model that explains Anfinrud’s stretched exponential conformational relaxation kinetics

At ambient temperatures, conformational relaxation in both myoglobin and hemoglobin occurs with a nonexponential time course. The most dramatic example of nonexponential relaxation kinetics is the work of Anfinrud and coworkers, who followed the kinetics of the shift of a conformationally-sensitive optical absorption band at 760 nm [33]. They observed the relaxation occurring from picoseconds to microseconds, which could be described by a stretched exponential (~ exp [-(*kt*)^β^]) with an extremely low stretching exponent of β ~ 0.1 (Fig. 5).

One of the interesting questions about nonexponential conformational relaxations is whether there is a theory to explain it. Thus far, only one theoretical model has been proposed [35]. The theory of Hagen et al*.* is attractive because it is based on a change in the conformational substate energies between the initial and final equilibrium conformations, Mb* and Mb respectively. To reproduce a stretched exponential time course, Hagen’s model requires the rather bold assumption of Koper and Hillhorst [36], as suggested by Frauenfelder et al*. *[16], that the initial protein conformational substates are connected to the final substates and to each other via transition states of a single energy *E*^‡^. A possible structural explanation for a single transition state energy suggested by Hagen et al. is that it corresponds to less compact conformations where the helices are slightly separated and free to reposition. The rate coefficient *k*_*ij*_ for a conformational substate of energy *E*_*i*_ in Mb* to convert to a new conformational substate in Mb with an energy between *E*_*j*_ and δ*E* is given by.

$${k_{ij}} = {k_0}g({E_j})\delta E\left[ {\exp \left( { - \left( {{E^\dag } - {E_i}} \right)/{k_B}T} \right)} \right]$$

where *k*_0_ is a constant and *g*(*E*_*j*_) is the density of states with energies between *E*_*j*_ and *δE*_*j*_, assumed for simplicity to have the same Gaussian distribution of substate conformations with energies *E*_*i*_ and *E*_*j*_. The highly extended time course arises from distributed kinetics: conformational substates with different ground state energies and therefore different activation energies and rate coefficients make the transition from Mb* to Mb via the transition state of energy *E*^‡^, after which the conformational substates of Mb slowly redistribute to achieve a Boltzmann distribution.

## The quaternary two-state allosteric model of MWC

In the kinetic models of Ansari et al. [21] and Hagen et al*.* [35], the conformation of Mb immediately after photodissociation was called Mb*. The asterisk was to draw an analogy with Hb*, the symbol given by Quentin Gibson for the quickly reacting form of hemoglobin in his microsecond CO photodissociation studies [37]. John Hopfield, Seiji Ogawa and Robert Shulman later showed that Gibson’s Hb* to Hb relaxation is actually the fast CO binding and high oxygen affinity R conformation transitioning to the slow CO rebinding and low oxygen affinity T conformation of the two-state allosteric model of Monod, Wyman, and Changeux (MWC) [2, 38]. The model was created by MWC to explain the observation that ligands, called allosteric effectors, alter enzyme activity by binding at a site distant from the catalytic site and proposed that it is a general mechanism for how enzymes perform the function of metabolic regulation. Hemoglobin, with more structural and biochemical information than any other protein at the time, became an “honorary enzyme” for allostery and was the major application of their model. The MWC model replaced the sequential model for cooperative oxygen binding by hemoglobin, proposed by Linus Pauling in his remarkable 1935 paper [39]. In Pauling’s model, binding of oxygen at one heme alters the affinity for oxygen of one or more hemes in neighboring subunits, so the affinity continues to increase with each binding step.

**Fig. 6:**
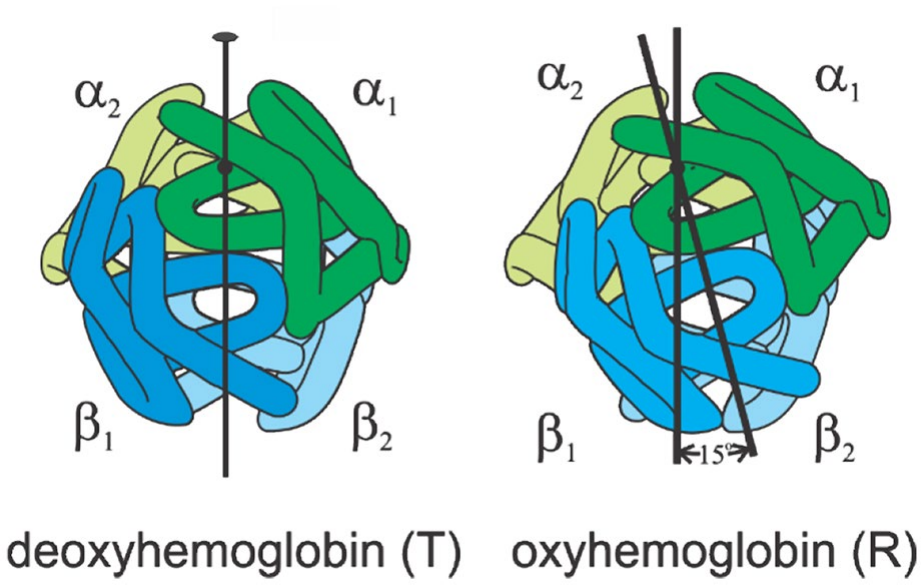
Schematic structures of deoxy and oxyhemoglobin showing that the main difference in structure corresponds to a rotation of 15 degrees of αβ pair of subunits relative to the other (adapted from Dickerson and Geis [3])

In the MWC model, there are 2 arrangements of its 4 subunits, called quaternary structures: a low oxygen-affinity T quaternary conformation corresponding to the structure of deoxyhemoglobin and a high oxygen-affinity R quaternary conformation, corresponding to the structure of oxyhemoglobin (Fig. 6). Binding to both quaternary conformations is perfectly non-cooperative; the sigmoid-shaped binding curve arises from a shift in the T-R equilibrium toward R as successive molecules of oxygen bind. Another key feature of the MWC model is that allosteric effectors do not alter the affinity of either T (*K*_*T*_) or R (*K*_*R*_). They only change the T-R equilibrium constant, *L* (= [T_0_]/[R_0_]). The elegantly simple MWC partition function for equivalent α and β subunits is.

$$\Xi {({\text{MWC)}}_{\alpha = \beta }} = L{\left( {1 + {K_T}p} \right)^4} + {\left( {1 + {K_R}p} \right)^4}$$

where *p* is the oxygen pressure. The saturation function, *y*(*p*), is given by

$$y(p) = \frac{1}{4}\frac{d\ln \Xi }{{d\ln p}}$$

**Fig. 7:**
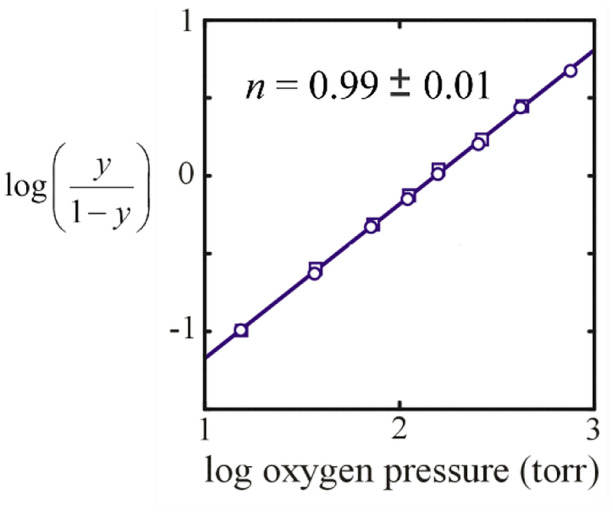
Hill plots of data for light polarized parallel to the a crystal axis showing that binding of the T conformation is non cooperative [47]. A slope of 1.0 in this kind of plot indicates no cooperativity. The slope for hemoglobin in solution is 2.8, indicating high cooperativity; the maximum value possible is the number of subunits, 4. The circles correspond to increasing oxygen pressure and the squares to decreasing oxygen pressure indicating that binding is at equilibrium. The line through the data is the least squares fits

The application of the MWC model to hemoglobin has had a long and controversial history. In subsequent studies, Gibson concluded that MWC is inconsistent with his microsecond resolved CO photolysis experiments, even taking into account the dependence of quaternary conformational rates on the number of CO molecules bound to the hemoglobin tetramer not considered by Hopfield et al. [40]. Moreover, at one point the MWC model applied to hemoglobin was almost completely discarded by most biochemists because of apparently convincing arguments of Gary Ackers using thermodynamic linkage relations connecting his tetramer-dimer dissociation results to oxygen binding [41, 42]. His work was later shown to be wrong because of experimental artifacts resulting from electron exchange that were not taken into account in his analysis [43], because of large differences between the directly measured oxygen binding curves by others and those predicted from his thermodynamic linkage analysis [44], and from experiments by Andrea Mozzarelli and coworkers described below. A key feature of the MWC model is that the T conformation binds oxygen non-cooperatively with cooperativity only arising from the T to R transition, as opposed to the conclusion of Ackers that the T conformation is highly cooperative. Non-cooperative binding to T was clearly demonstrated by Mozzarelli and colleagues using a microspectrophotometer to measure oxygen binding curves for single crystals of hemoglobin known from X-ray crystallographic studies to remain in the T quaternary structure with oxygen bound (Fig. 7) [45–47].

**Fig. 8:**
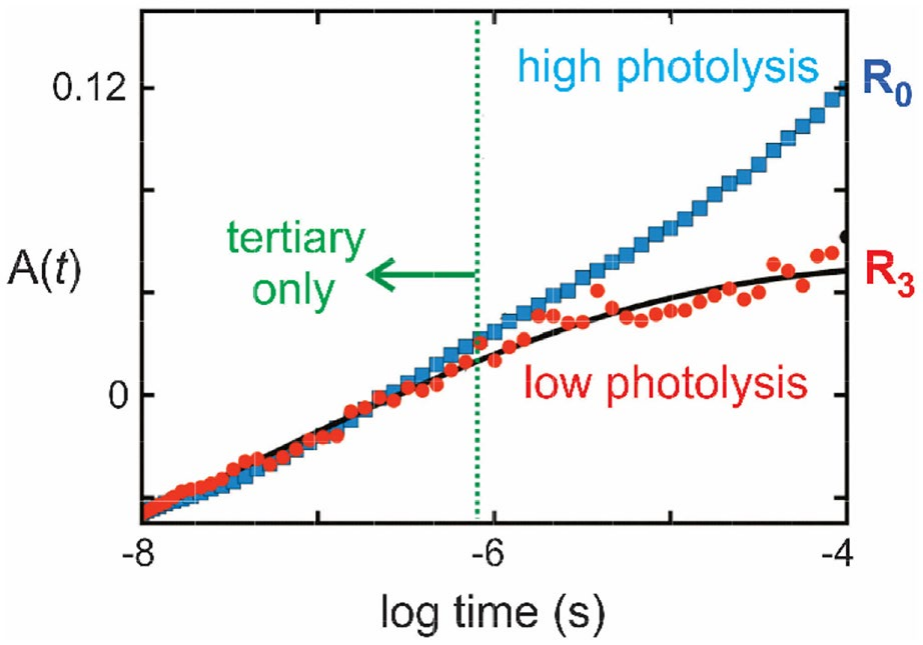
Distinguishing tertiary and quaternary protein conformational changes. Data from ref. [49] Amplitude of the 2^nd^ SVD component as a function of time following photodissociation of the CO complex of hemoglobin. The 2^nd^ SVD component represents the spectral change of the unliganded hemes corresponding to the protein conformational change. At low photolysis the only populated photodissociated species is three-fold liganded R (R_3_), which is thermodynamically stable and does not convert to T_3_. Consequently, the red points correspond to a pure tertiary conformational relaxation with a stretching exponent of β = 0.2

Hopfield et al. went further to show more definitively that the MWC model explained a wide variety of equilibrium results from NMR and kinetics results from the experiments of Eraldo Antonini, Maurizio Brunori, and others, as well as Gibson’s [38, 48]. To simplify the modeling, Hopfield et al. assumed that all conformational transitions are so rapid, compared to oxygen and carbon monoxide binding and dissociation, that they can be treated as being at constant equilibrium. The next advance came from the nanosecond-resolved absorption spectroscopic studies of Hofrichter et al. [12]. Application of a sophisticated version of SVD by Eric Henry to the transient spectra measured with Hofrichter’s instrument permitted the time course of the deoxyheme spectral change due to the protein conformational change to be measured with high signal-to-noise [12]. A comparison of the kinetics of the spectral change at full photolysis and partial photolysis, where only fully (R_4_) and threefold liganded hemoglobin molecules (R_3_) are populated, which remain in the R conformation, permitted acquisition of the time course of the hemoglobin tertiary conformational relaxation (Fig. 8) [49]. As with myoglobin, it is a highly stretched exponential with a stretching exponent of β=0.2, which could very well turn out to be much smaller once it is measured with picosecond time resolution. By adding the stretched exponential tertiary conformational relaxation and a linear free energy relation between quaternary rate coefficients and equilibrium constants, it became possible to quantitatively explain ligand binding and protein conformational changes with a kinetic model consistent with MWC [50, 51].

## The tertiary two-state allosteric model of Henry et al*.*

**Fig. 9:**
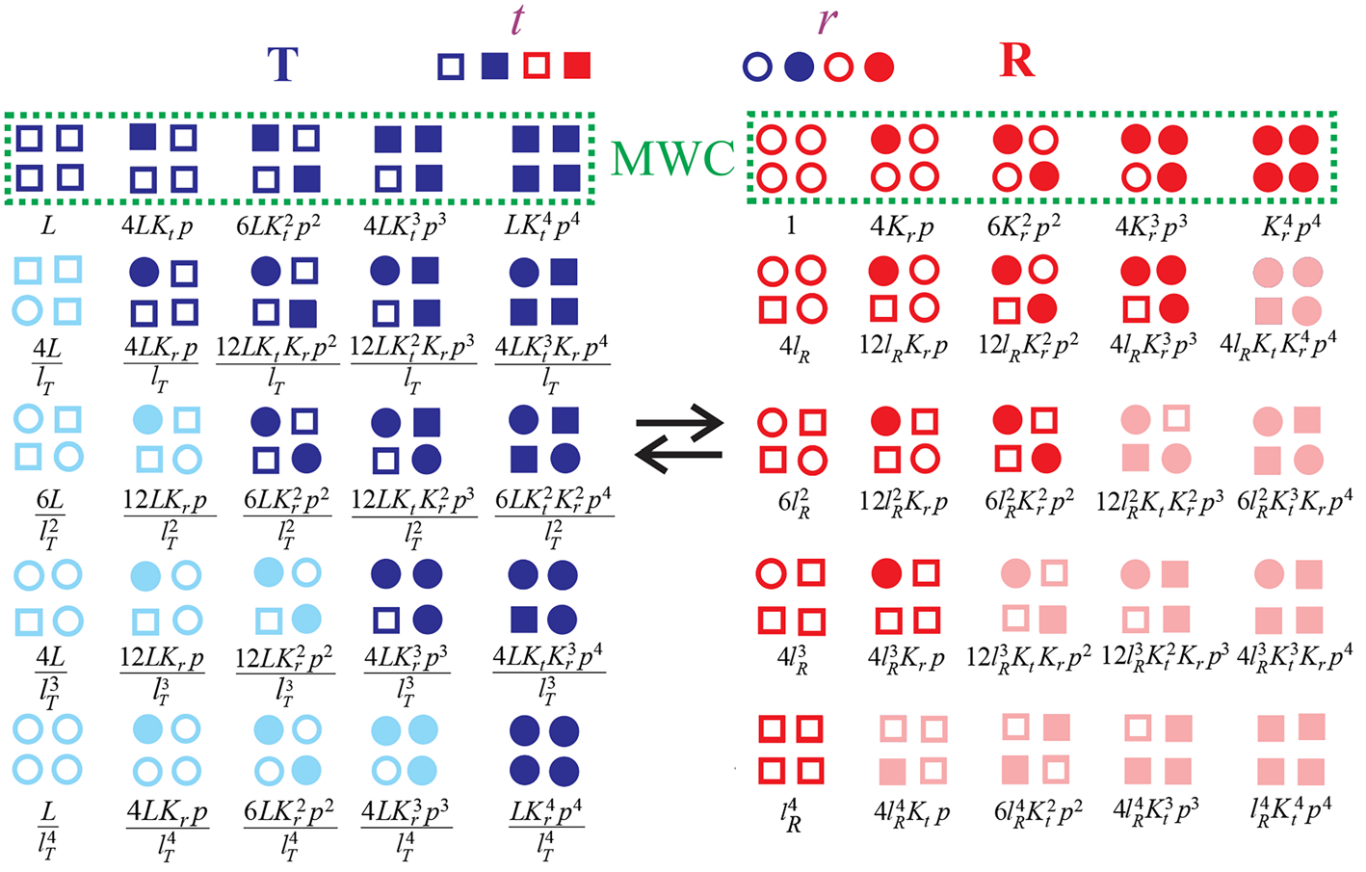
Diagrammatic representation of MWC and TTS models with Boltzmann weights for the 50 terms of the TTS partition function. Unliganded subunits are represented by open symbols and liganded subunits by filled symbols. Conformations in light color have a very low probability. See ref. [32] for a more detailed description

The observation and characterization of tertiary conformational kinetics led to the development by Henry et al*.* of the simplest extension of the quaternary two-state MWC model to include tertiary conformational changes (the tertiary two-state (TTS) model) shown schematically in Fig. 9 [52]. The TTS model was motivated in part by the observation in our nanosecond-resolved photolysis studies that the spectral changes for both tertiary and quaternary conformational changes are the same. According to the TTS model, two tertiary conformations, called *t* and *r*, exist in each quaternary structure and have the same equilibrium and kinetic properties in both. The partition function for this model is [52]

$${\Xi }{({\text{TTS}})_{\alpha = \beta }} = \frac{L}{{{{\left( {l_T} \right)}^4}}}{\left[ {1{ + }K_rp + l_T\left( {1 + K_tp} \right)} \right]^4} + {\left[ {1{ + }K_rp + l_R\left( {1 + K_tp} \right)} \right]^4}$$

where *L* is the T/R population ratio, in which all the subunits of T are unliganded *t* and all the subunits of R are unliganded *r*, *l*_*T*_ is the *t/r* tertiary population ratio of unliganded subunits in the T quaternary structure, and *l*_*R*_ is the *t*/*r* tertiary population ratio of unliganded subunits in the R quaternary structure. *K*_*t*_ is the ligand-binding equilibrium constant for *t*, *K*_*r*_ is the ligand binding equilibrium constant for *r*, and *p* is the oxygen or CO pressure. In the MWC model, allosteric effectors change only *L*, whereas in the TTS model they can change all three conformational equilibrium constants, *L*, *l*_*T*_, and *l*_*R*_. The above partition function is valid at constant pH without effector ligands or with saturating effector concentrations, as in our experiments described below, assuming that the effector-binding free energy is linearly proportional to the fraction of *t* or *r* subunits [53].

**Fig. 10:**
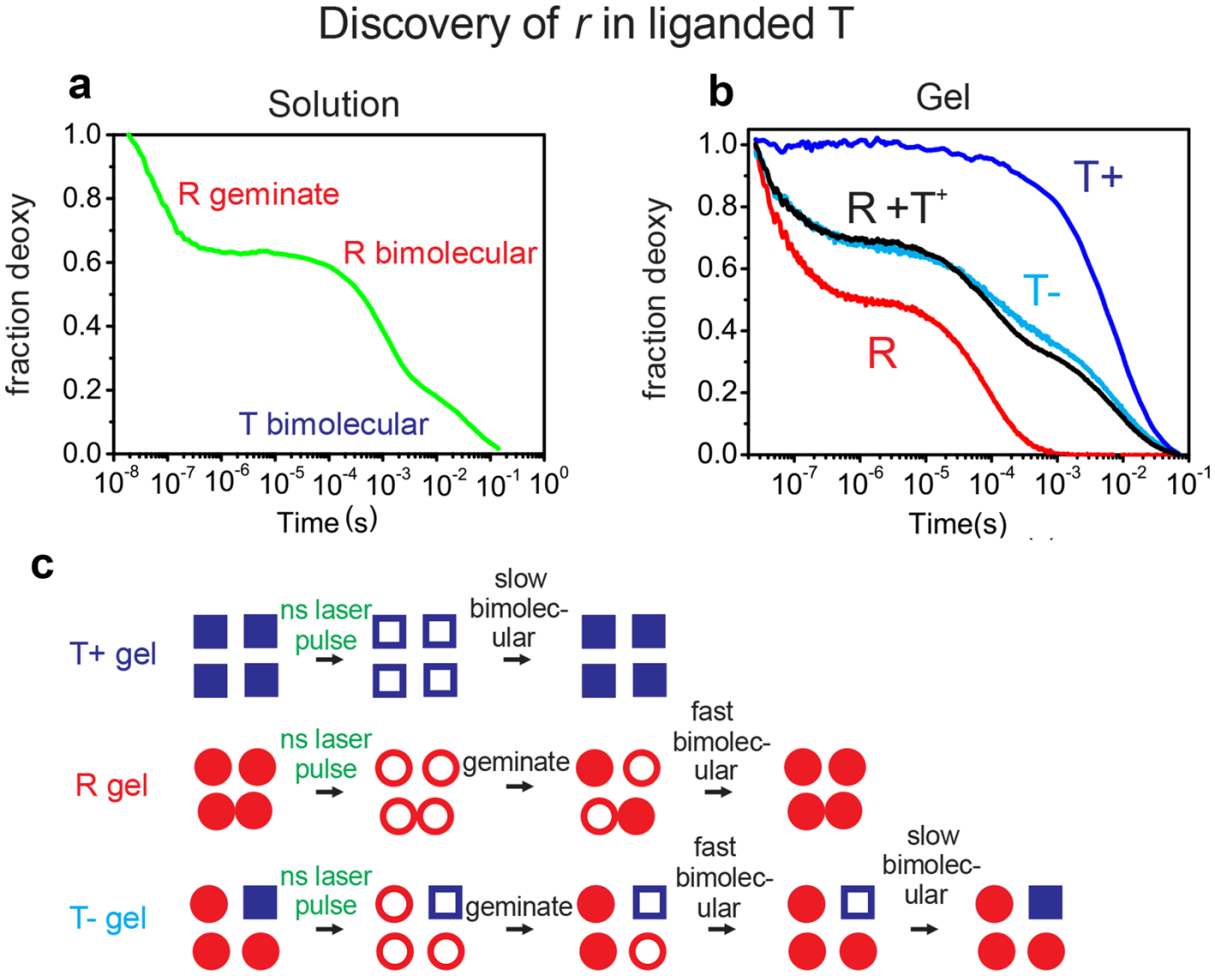
Results and explanation of CO nanosecond photolysis experiments of hemoglobin encapsulated in silica gels [55]. (**a**) three phases of CO rebinding following photodissociation of hemoglobin CO complex in solution. (**b**) CO rebinding kinetics following photodissociation of the R and T conformations encapsulated in silica gels saturated with allosteric effectors (T + , blue curve) and with no allosteric effectors (T-, cyan curve). The geminate rebinding amplitude in R is larger in the gel than in solution because there is no tertiary relaxation (r* to *r*) on the sub-second time scale in the gel to slow geminate rebinding. The fraction of *r* subunits in T- shown in panel c is obtained from the optimal linear combination of R and T + (black curve). (**c**) Explanation in terms of TTS model. Blue squares correspond to the *t* tertiary conformation, unfilled squares for unliganded and filled squares for liganded. Red circles correspond to the *r* tertiary conformation, unfilled circles for unliganded and filled circles for liganded

Most importantly, the model explains the remarkable findings in nanosecond photolysis experiments by Cristiano Viappiani, Mozzarelli, and coworkers, in which encapsulation of hemoglobin in silica gels traps deoxyhemoglobin in a single quaternary conformation, as well as in unstable tertiary conformations [54]. In the first set of experiments CO-rebinding subunits were discovered in the T quaternary conformation in the absence of allosteric effectors (T-) that have the same fast rebinding rate as in the R quaternary structure; according to the TTS model these fast-rebinding subunits in T are in the *r* tertiary conformation (Fig. 10). No *r* subunits exist in liganded T at saturating concentrations of allosteric effectors (T+) [55].

**Fig. 11:**
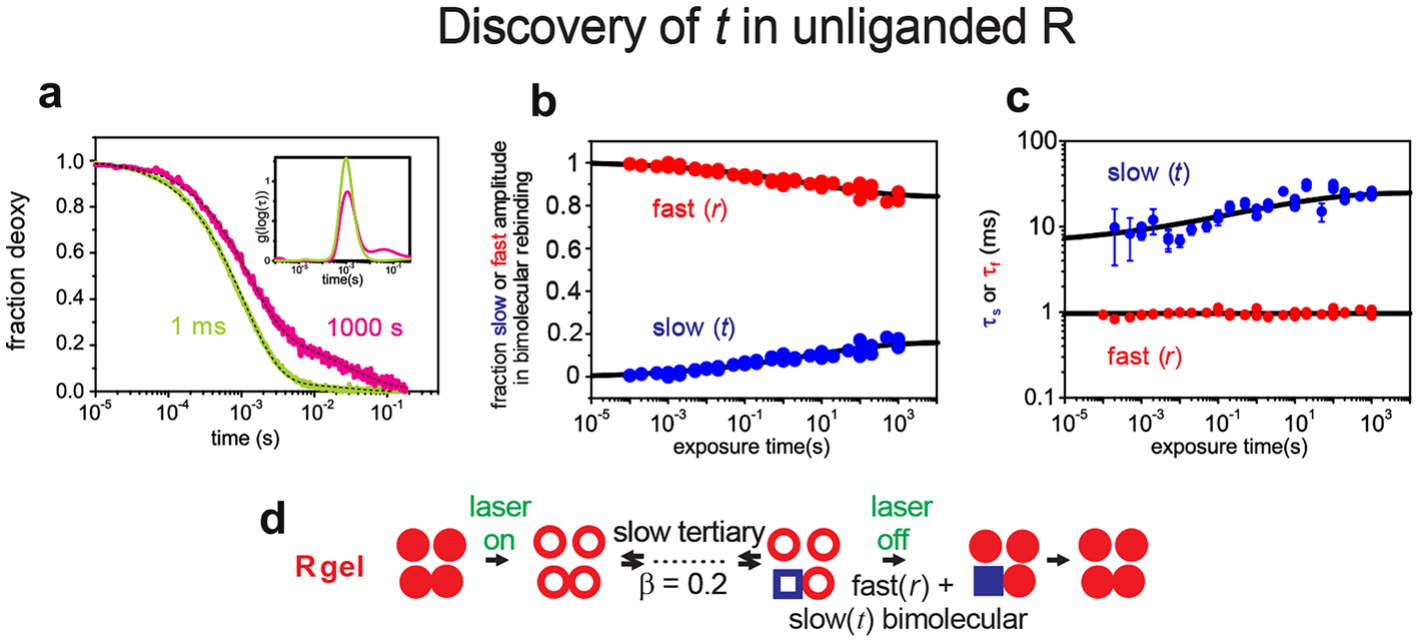
Results and explanation of nanosecond-pulsed/cw CO photolysis experiments of R hemoglobin encapsulated in a silica gel [56]. (**a**) Bimolecular rebinding following ns photolysis followed by 1 ms of cw photolysis to maintain the unliganded state (yellow-green curve) and by 1000 s of cw photolysis (pink curve) showing emergence of a slow rebinding phase without conversion to T. Inset shows rate distribution from a maximum entropy analysis. (**b**) Fraction of fast and slow bimolecular rebinding phases as a function of exposure time to cw photolysis. (**c**) Rebinding times as a function of exposure time to cw photolysis. (**d**) Interpretation of experiment in terms of TTS model. See interpretation of symbols in legend to Fig. 10c. The slow rebinding time increases slightly with exposure time because the ensemble of substates of *t* in R require the longer exposure times to fully equilibrate. The curves in panels b and c are stretched exponentials with a β = 0.2

In an even more demanding second set of experiments, CO was held off after pulsed photolysis of the R conformation with a cw laser for a sufficiently long time to allow tertiary conformations to relax to their equilibrium conformation (Fig. 11). The experiment showed that the deoxy R quaternary structure contains a fraction of subunits that rebind with T rates, and are therefore in the *t* tertiary confirmation – a key prediction of the TTS model, which is not anticipated by any other model [56]. 

The overall findings of the gel experiments are that Viappiani et al. discovered a fraction of the subunits in the *r* tertiary conformation in the liganded T quaternary structure and a fraction of the subunits in the *t* tertiary conformation in the unliganded R quaternary structure. Interestingly, according to Perutz, salt bridges are responsible for the low affinity of T and break when ligands bind [57]. Consequently the partition function of Attila Szabo and Martin Karplus describing the Perutz stereochemical predicts that all subunits trapped in the liganded T conformation should show R-state rebinding kinetics [53]. In the Viappiani experiments only a fraction shows R-state rebinding kinetics because both *t* and *r* are populated in liganded T. Neither the Szabo-Karplus model nor any other model other than TTS predicts T-state ligand binding kinetics to R.

A comparison of the TTS model with other proposed models for hemoglobin cooperativity shows that it is the only model capable of explaining the results on hemoglobin in crystals and silica gels [32]. However, hemoglobin is a complex system and the TTS model is not quantitatively perfect. It must therefore be oversimplified, one example being the lack of inclusion of more than one R-quaternary structure in kinetic models [58]. Nevertheless, like the MWC model, the TTS model should motivate new experiments and theoretical investigations that will result in an even more accurate model for allostery in hemoglobin and multi-subunit proteins in general. A major outstanding problem for understanding the function of hemoglobin is to provide an atomistic structural explanation for the 1000-fold difference in oxygen affinity between the *t* and *r* subunits [32, 52]. An atomistic explanation was first attempted by Max Perutz in his groundbreaking paper on the difference of R and T affinity and the origin of the Bohr effect, with the corresponding partition function developed by Szabo and Karplus, that marked the beginning of the investigation of detailed structure–function relations in proteins [53, 57]. Structures are known for unliganded and liganded *t* in T and *r* in liganded R, but a complete structural explanation will have to await the determination of the structures of *r* in fully liganded T and *t* in fully unliganded R. An even more challenging problem is the structural explanation for the 2,500-fold difference in the initial geminate rebinding rate for conformationally unrelaxed (Mb*(conf) = MbCO(conf)) and conformationally relaxed (Mb(conf)) myoglobin, considering that conformational differences between myoglobin and its CO complex are barely detectable by X-ray crystallography (see Fig. 1 in ref [21]).

## Concluding Comments

My short story has shown how the modern view of protein reaction kinetics introduced by Hans Frauenfelder and Peter Wolynes motivated my research group, as well as many others, to think about and study both myoglobin and hemoglobin in new ways. The results have been a series of novel and important findings, which have added to their pioneering work and have greatly improved our understanding of the function of both the hydrogen atom and hydrogen molecule of biology. However, even after over 100 years of biophysical research on hemoglobin [59], important problems remain to be solved by physicists and physical chemists.

## Data Availability

All data presented here has been published in papers by author. References are provided.
